# Tangent field technique of TomoDirect improves dose distribution for whole‐breast irradiation

**DOI:** 10.1120/jacmp.v16i3.5369

**Published:** 2015-05-08

**Authors:** Harumitsu Hashimoto, Motoko Omura, Kengo Matsui, Yuki Mukai, Hideyuki Hongo, Wataru Yamakabe, Kaori Saito, Miwa Yoshida

**Affiliations:** ^1^ Departments of Radiology and Shonan Kamakura General Hospital Kamakura Kanagawa Japan; ^2^ Radiation Oncology Shonan Kamakura General Hospital Kamakura Kanagawa Japan; ^3^ Department of Radiology Shonan Fujisawa Tokushukai Hospital Fujisawa Kanagawa Japan; ^4^ Department of Radiology Yokohama City University Graduate School of Medicine Yokohama Kanagawa Japan

**Keywords:** TomoDirect, IMRT, tangent field technique, breast cancer, lung dose

## Abstract

TomoDirect (TD) is an intensity‐modulated radiotherapy system that uses a fixed gantry angle instead of rotational beam delivery. Here, we investigated the effect of the multiple beam technique of TomoDirect on dose distribution compared with commonly‐used tangential beams. We included 45 consecutive patients with right breast cancer who underwent postoperative radiotherapy in our institute in the present study. Clinical target volume (CTV) was the whole right breast. The planning target volume (PTV) was created by expanding the CTV by a 0.5 cm margin. Paired TD plans were generated for each patient; a two‐beam plan using paired tangential beams and a six‐beam plan with four additional beams with modified gantry angles of $\pm \;5^{{}^{\circ}}$ from the original tangential beam set. A prescribed dose of 50 Gy was defined for 50% isodoses of the PTV. The six‐beam plan delivered significantly more homogeneous doses to the PTV than the two‐beam plan; and the mean dose to the PTV in the six‐beam plan more closely reflected the prescribed dose. $V_{20\text{Gy}}$ and mean dose to the right lung and mean dose to the whole body were also significantly decreased in the six‐beam plan. However, duration of radiation exposure was 1 min longer in the six‐beam plan than in the two‐beam plan. The dose distribution to the target and organs at risk were improved with the six‐beam plan relative to the two‐beam plan without increasing the whole‐body radiation dose. The six‐beam plan using TD is a simple technique that can be routinely applied to whole‐breast irradiation in clinical practice.

PACS number: 87.55

## I. INTRODUCTION

The TomoTherapy System has been developed for intensity‐modulated radiation therapy (IMRT). TomoHelical, an original system of the TomoTherapy, provides rotational delivery of a fan beam, such as that generated by a computed tomography (CT) scanner.^1^, ^2^ The TomoDirect (TD) is a new type of TomoTherapy IMRT system that uses a fixed gantry angle instead of rotational beam delivery.^3^, ^4^

The effects of intrafraction respiratory motion on IMRT have been reported, showing that it can induce the unacceptable deviations between planned and delivered dose.^5^, ^6^ In contrast, the TD system has a unique function designed to compensate for those effects. It provides a one to five collimator leaf expansion function at the anterior edge of the beams, which helps deliver the exact prescribed dose to the target by accounting for movement during irradiation, such as breathing.^(7)^ This technique is thus appropriate for whole‐breast irradiation. In our institute, we used TD for whole‐breast irradiation after conservative surgery for breast cancer.

In TD plans, even when only tangential direction beams (paired tangential beams) are used, dose distribution to breast tissue is superior to conventional three‐dimensional conformal radiation therapy.^(4)^ Whilst the use of multiple direction beams might further improve the dose distribution to the target, it could also increase the volume of the surrounding normal tissue, including the lung exposed to lower dose radiation.^(4)^ Based on our clinical experience, we thought that in multiple beam techniques using TomoDirect, a careful arrangement of the beam angles would be essential to achieving a better dose distribution. In this study we evaluated whether the TD multiple‐beam technique could balance dose distributions to targeted tissue while sparing normal tissue in whole‐breast irradiation, compared with original tangential beams.

## II. MATERIALS AND METHODS

### A. Patients and image acquisition

We included 45 consecutive patients with primary cancer in their right breasts in this planning study. They underwent breast‐conserving surgery and postoperative radiation therapy in our institute between October 2010 and September 2012. Their median age was 60 years old (range: 28–79 yrs). Only patients with right breast cancer were included in this study to control for the influence of heart volume on lung dose. Written informed consent, for both participation in this study and its publication (with identifying personal information concealed), was obtained from each patient before treatment. Treatment planning CT images were obtained by LightSpeed Ultra 8 Slice (General Electric Healthcare, Pewaukee, WI). Patients were instructed to lie in the supine position with their arms raised above their head using an arm support and to breathe naturally. The CT images were taken at 2.5 mm intervals and were transferred to Pinnacle^3^ ver. 9.0 (Philip Medical System, Eindhoven, The Netherlands).

### B. Volume contouring

**Figure 1:**
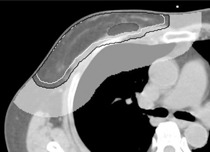
Contouring of a representative case. The clinical treatment volume (CTV) included the whole right breast, and was expanded 5 mm in the anterior–posterior and left–right directions to create a planning treatment volume (PTV) that excluded a 3 mm strip of skin and the lung tissue. The PTV ring was delineated around the PTV at 3 cm. Black mesh=tumor bed;white line=CTV;black line=PTV;white mesh=PTV ring.

All contouring of target volume and normal structure were obtained using the Pinnacle^3^. The clinical target volume (CTV) was defined as the whole right breast, including the remaining mammary glandular tissue. The CTV was expanded 5 mm in the anterior–posterior and left–right directions to create a planning target volume (PTV) that excluded a 3 mm strip of skin and the lung tissue. Additionally, the tumor bed was defined by referring to preoperative CT images, operative notes, and surgical wounds. The left breast and right lung were identified as organs at risk (OARs). The PTV ring was delineated around the PTV at 3 cm to suppress the dose outside the PTV (Fig. 1). The CT images and volume contours were transferred to the TomoTherapy System (Accuray, Sunnyvale, CA).

### C. Planning

A TD plan using tangential beams (two‐beam plan) was generated for each patient by TomoPlan ver. 4.0. The paired tangential beam angles were determined based on a technique for a conventional linear accelerator. Those beam angles were arranged to include PTV and to minimize doses to OARs, ipsilateral lung and contralateral breast. The entire breast tissue and chest wall were included in the irradiation volume.

**Figure 2:**
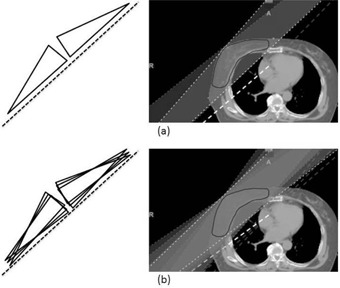
Beam directions of two‐beam (a) and six‐beam (b) plans, with $\pm \;5^{{}^{\circ}}$ beams added to the original tangential beams of two‐beam plan.

A six‐beam plan was then generated that contained an additional four beams with modified gantry angles of $\pm \;5^{{}^{\circ}}$ from the original tangential beam set (Fig. 2). The reason to choose $\pm \;5^{{}^{\circ}}$ was that this beam angle was thought to maximally reduce doses to ipsilateral lung and contralateral breast tissue. None of the beams were directly opposed.

Initially, the same parameters for dose calculation were used for both plans and then modified during plan optimization. The field width and pitch were 2.5 cm and 0.25, respectively. The modulation factor was 2.0. The grid resolution for final dose calculation was 0.2 cm.

### D. Dose prescription and constraint

To allow for comparison, the same dose prescription for targets and constraints for OARs were used for both plans. A prescription of 50 Gy was defined for 50% isodoses of the PTV in 25 fractions of 2.0 Gy daily. The PTV volume (95%) should receive at least 95% of the prescribed dose (47.5 Gy).

Dose constraints for OARs were a) right lung: $V_{20\;\text{Gy}}<20\%;b$) left beast: Dose max <5 Gy, with mean dose as low as possible; c) body: Dose max <55 Gy, 110% of the prescribed dose; and d) PTV ring: mean dose as low as possible. Left lung and heart were not estimated as no planned beams entered or exited through those organs in most cases and their dose distributions were negligible. Dose‐volume histogram points and penalties were adjusted throughout the optimization to best meet OAR dose constraints without compromising the PTV coverage mentioned above.

### E. The plan assessment

The following parameters were compared between the two‐beam plan and the six‐beam plan: a) minimum dose of radiation to the tumor bed; b‐1) PTV homogeneity index (HI, the difference in the doses that cover 95% and 5% of the PTV, which represents the dose homogeneity of the target volume); b‐2) mean dose of PTV; b‐3) PTV $V_{95\%}$, (percentage of target volume that receives 95% of prescription dose, 47.5 Gy), PTV $V_{100\%},V_{105\%}$, and $V_{107\%}$; c‐1) right lung $V_{5\;\text{Gy}}$, (subvolume that receives 5 Gy) and right lung $V_{10\text{Gy}}$, $V_{15\text{Gy}}$, $V_{20\text{Gy}}$, $V_{30\text{Gy}}$, and $V_{40\text{Gy}}$; c‐2) mean dose of radiation to the right lung; d) PTV ring $V_{45\text{Gy}}$ and its mean dose (which indicate the dose concentration to PTV and the limitation of dose to normal surrounding tissue); e) maximum dose of radiation to the left breast; f) mean dose of radiation to the whole body; and g) duration of radiation exposure (which indicates approximate treatment time and workload of the medical equipment).

Each parameter for the two plans was statistically analyzed using a paired two‐sided Wilcoxon‐Mann‐Whitney test; p<0.01 was considered statistically significant.

## III. RESULTS

Dose‐volume data for these two plans are summarized in Table 1 and shown in 3, 4. All variables significantly differed between the two plans, except for minimum dose to the tumor bed (Table 1) which reached 48.5 Gy, more than 95% of the prescription dose in both plans. The six‐beam plan showed better homogeneity of dose distribution for PTV (HI) than the two‐beam plan. Mean dose to PTV in the six‐beam plan more closely reflected the prescribed dose of radiation (50 Gy) than did the two‐beam plan. Additionally, PTV $V_{95\%}$ $V_{100\%}$, $V_{105\%}$, and $V_{107\%}$ were better controlled and closer to the prescribed dose in the six‐beam plan. This result indicated that the six‐beam plans can deliver radiation more effectively to a PTV, with more precise dosing and less excess radiation, than do the two‐beam plans, which delivered unnecessary radiation to the same PTVs.

**Figure 3:**
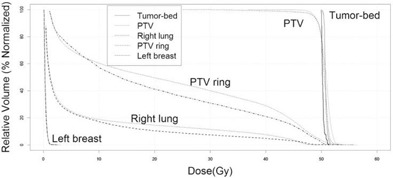
Comparison of dose‐volume histograms in two‐beam plan and six‐beam plan of a representative case. In the six‐beam plan, PTV more closely refected the prescribed dose of radiation (50 Gy) The right lung subvolume that received >15 Gy was decreased, but not the subvolume that received the lower dose (<10 Gy). Dose to the PTV ring was suppressed. Thin lines=two–beam plan;thick lines=six–beam plan.

**Figure 4:**
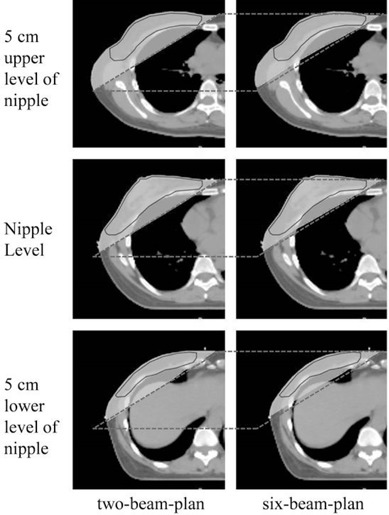
Comparison of dose distributions in two‐beam plan and six‐beam plan of a representative case. The area that received a dose of 20 Gy included more lung tissue in the two‐beam plan than in the six‐beam plan. Black lines=PTV; gray shaded area=20 Gy zone; oblique side of gray parallelogram=tangential beams angle.

**Table 1:** Evaluation parameters for two‐beam and six‐beam plans.

	*Parameters*	*Unit*	*Two‐beam Plan* (mean±SD)	*Six‐beam Plan* (mean±SD)	*p‐value*
Tumor bed	Minimum dose	Gy	50.16±0.80	50.19±0.65	0.87
PTV	HI	Gy	4.02±0.73	3.75±0.62	<0.01
	Mean dose	Gy	50.66±0.30	50.36±0.21	<0.01
	$V_{95\%}$	%	97.13±1.18	96.38±0.83	<0.01
	$V_{100\%}$	%	78.87±5.32	75.72±4.72	<0.01
	$V_{105\%}$	%	6.19±6.71	1.68±2.25	<0.01
	$V_{107\%}$	%	0.81±1.58	0.09±0.23	<0.01
Right lung	$V_{5\text{Gy}}$	%	25.41±5.99 ^a^	28.21±7.54 ^a^	<0.01
	$V_{10\;\text{Gy}}$	%	20.41±5.91 ^a^	22.00±7.17 ^a^	<0.01
	$V_{15\;\text{Gy}}$	%	17.96±5.76	16.43±5.22	<0.01
	$V_{20\;\text{Gy}}$	%	16.09±5.57	13.45±4.56	<0.01
	$V_{30\;\text{Gy}}$	%	12.77±5.06	10.25±4.19	<0.01
	$V_{40\;\text{Gy}}$	%	9.21±4.50	7.57±3.97	<0.01
	Mean dose	Gy	8.16±2.40	7.72±2.31	<0.01
PTV ring	$V_{45\text{Gy}}$	%	18.67±4.90	17.03±6.19	<0.01
	Mean dose	Gy	22.07±3.21	20.86±3.10	<0.01
Left breast	Max dose	Gy	1.47±0.97 ^b^	2.21±1.53 ^b^	<0.01
Whole body	Mean dose	Gy	4.00±0.88	3.91±0.87	<0.01
Duration of radiation exposure	Time	min	4.9±0.6 ^b^	5.6±0.5 ^b^	<0.01

^a^ The area that received a 20 Gy dose included more lung tissue in the two‐beam plan than in the six‐beam plan, whereas in the six‐beam plan, right lung $V_{5\text{Gy}}$ and $V_{10\text{Gy}}$ were increased instead.

^b^ Maximum radiation dose to the left breast and duration of radiation exposure increased in the six‐beam plan.

HI=homogeneity index (difference of dose between 95% and 5% of the PTV); PTV $V_{95\%},V_{100\%},V_{105\%}$, and $V_{107\%}=\text{PTV subvolumes}$ that receive 47.5 Gy, 50 Gy, 52.5 Gy, and 53.5 Gy, respectively; Right lung $V_{5\text{Gy}}$, $V_{10\text{Gy}}$, $V_{15\;\text{Gy}}$, $V_{20\;\text{Gy}}$, $V_{30\;\text{Gy}}$, and $V_{40\;\text{Gy}}=\text{the right lung subvolumes that receive}$ 5, 10, 15, 20, 30, and 40 Gy, respectively; PTV ring $V_{45\text{Gy}}=\text{the PTV}$ ring volume that receives 45 Gy.

Compared with the two‐beam plan, right lung $V_{15\text{Gy}}$, $V_{20\text{Gy}}$, $V_{30\text{Gy}}$, $V_{40\text{Gy}}$ and its mean dose in the six‐beam plan were significantly decreased (Table 1). Figure 3 compares dose‐volume histograms in two‐beam plan and six‐beam plan of a representative case. The area that received a 20 Gy dose included more lung tissue in the two‐beam plan than in the six‐beam plan, whereas in the six‐beam plan, right lung $V_{5\text{Gy}}$ and $V_{10\text{Gy}}$ were increased instead (Fig. 3 and Table 1 (as noted)). The PTV ring $V_{45\text{Gy}}$ and its mean dose were decreased in the six‐beam plan (Fig. 3 and Table 1).

Finally, mean radiation dose to the whole body were decreased in the six‐beam, although maximum radiation dose to the left breast and duration of radiation exposure increased in the six‐beam plan (Table 1 as noted).

## IV. DISCUSSION

The present study evaluated two TD treatment plans for whole‐breast irradiation. Those plans used tangential directions beams (two‐beam plan), and six beam directions that added $\pm \;5^{{}^{\circ}}$ beams to the original two beam (six‐beam plan). We found that the dose distributions to the target area and OARs with the six‐beam plan were superior to the two‐beam plan without increasing the mean whole‐body radiation dose. This is one of the few reports to describe the benefit of multiple‐beam application for whole‐breast irradiation using TD.^3^, ^4^

When comparing the six‐beam plan to the two‐beam plan, PTV dose homogeneity was significantly improved, and its mean values were closer to the prescribed doses. Additionally, $V_{95\%},V_{100\%},V_{105\%}$, and $V_{107\%}$ of PTV were better controlled in the six‐beam plan. This result indicates that six‐beam plans deliver radiation more effectively and precisely to PTVs, whereas the two‐beam plan delivered excess radiation to achieve the PTV dose coverage. In particular, $V_{107\%}$ was much lower in the six‐beam plan than the two‐beam plan, which indicated that the six‐beam plan could reduce excess dose distribution.

With respect to right lung dose, mean dose and indexes, except for $V_{5\text{Gy}}$ and $V_{10\text{Gy}}$, were less in the six‐beam plan than in the two‐beam plan. As the risk of radiation‐induced pneumonitis correlates with lung volume that receives >20 Gy and with mean dose, its risk may be decreased in the six‐beam plan.^8^, ^9^

The $V_{45\text{Gy}}$ and mean dose to the PTV ring (i.e., normal tissue surrounding PTV) were significantly less in the six‐beam plan than in the two‐beam plan. The decreased PTV ring $V_{45\text{Gy}}$ indicates that the concentricity of the dose to the target improves. Simultaneous reduction of its mean dose indicates that dose distribution to the normal surrounding tissue also decreases. Moreover, the mean whole‐body radiation dose was lower in the six‐beam plan than in the two‐beam plan. To achieve the prescription dose distribution for PTV, less radiation exposure was necessary with the six‐beam plan than in the two‐beam plan.

Several reports have indicated that TD is suitable for postoperative whole‐breast irradiation, and have described two‐ or four‐beam plans that conformed to the PTV.^7^, ^10^, ^11^ Because patients with breast cancer on either or both sides were included in most references, comparing ipsilateral lung doses in those studies directly with the present study was difficult. Reynders et al.^(4)^ described a six‐field plan that contained tangential beams and two more sets of beam angles selected at 10°–20° from the initial tangential beams. They analyzed each side of breast cancer patients separately. For patients with the right‐side disease, although the six‐field plan improved dose distribution to PTV, the right‐lung dose equivalent to $V_{20\text{Gy}}$ increased to nearly twice that of the tangential field plan. In contrast, our plan used multiple beams with narrower angles (±5°), which improved PTV dose distribution and decreased right‐lung mean dose and $V_{20\text{Gy}}$. In addition, compared with those studies^7^, ^10^, ^11^ the maximum dose to the contralateral breast was sufficiently suppressed in the present study.

Several factors may have contributed to better dose distribution in the six‐beam plan than in the two‐beam plan. First, the six‐beam plan obviously provides greater flexibility for intensity modulation of dose delivery using multiple beams. Second, appropriately fitted tangential angles to the PTV gradually changed in each cross section vertically in the whole breast along with the curve of the patients' thorax. In the two‐beam plan, directions of beams were fixed tangential to the PTV in a representative cross section of the patient's CT images and could not fit the PTV in other sections. In contrast, the six‐beam plan contained additional beams at $\pm \;5^{{}^{\circ}}$ angles, and the combination of those multiple beams created a field that better fitted the PTV shape of each section of the whole breast. We added $\pm \;5^{{}^{\circ}}$ beams to the original two‐beam plans to create six‐beam plans for all cases in this planning study. However, in clinical practice, we can arrange the beam angles to fit the shape of each patient's body, based on this six‐beam plan, and obtain a better tailored dose distribution. As Asian patients have smaller breasts, $\pm \;5^{{}^{\circ}}$ beam angles were suitable for most of our patients, whereas larger beam angles (8°–10°) are appropriate for patients with larger breasts.

If more than six beams were applied, dose distribution to targets and dose sparing for OARs may be improved. However, treatment time could be also longer and might not be acceptable for clinical use. Our clinical experience indicates that duration of radiation exposure should be restricted to no more than 10 min, considering patients' stability and load on the machine. Mean radiation exposure in the six‐beam plan was 5.6 min, which was approximately 1 min longer than that of the two‐beam plan. This duration is acceptable for clinical use.

Some studies suggest that younger patients with breast cancer who are irradiated with tangential fields have greater risk of contralateral breast cancer, especially patients with a family history of breast cancer.^12^, ^13^ Another study reported a correlation between radiation dose to the contralateral breast and risk of developing breast cancer in both younger patients and those with gene mutations related to carcinogenesis.^(14)^ To date, dose constraints against contralateral breast cancer have not been established. Therefore, minimizing radiation delivered to the contralateral breast as much as possible is important. We found that the main disadvantage of the six‐beam plan was an increased maximum radiation dose to the left breast (2.21±1.53 Gy) relative to the two‐beam plan (1.47±0.97 Gy). However, retrospective studies of contralateral breast cancer development after radiation therapy indicate that 2.21 Gy might a sufficiently safe dose to the contralateral breast in terms of inducing malignancies.^(11)^ In addition, compared with previous planning studies that used IMRT, the maximum dose to the contralateral breast was sufficiently suppressed, even in six‐beam plan in this study.^4^, ^7^, ^10^, ^11^, ^15^

## V. CONCLUSIONS

In this planning study for whole‐breast irradiation using the TD system, dose distribution to target tissue and OARs in the six‐beam plan was superior to that of the two‐beam plan, without increasing the whole radiation dose. The six‐beam technique is simple and can be routinely applied to whole‐breast irradiation.

## ACKNOWLEDGMENTS

We thank the staff of the Department of Radiation Oncology at Shonan Kamakura General Hospital, and Dr. Hisato Nagano of Shonan Fujisawa Tokushukai Hospital, for their continued assistance.
